# Enrichment of Conserved Synaptic Activity-Responsive Element in Neuronal Genes Predicts a Coordinated Response of MEF2, CREB and SRF

**DOI:** 10.1371/journal.pone.0053848

**Published:** 2013-01-31

**Authors:** Fernanda M. Rodríguez-Tornos, Iñigo San Aniceto, Beatriz Cubelos, Marta Nieto

**Affiliations:** 1 Centro Nacional de Biotecnología (CNB-CSIC), Darwin 3, Campus de Cantoblanco, Madrid, Spain; 2 Facultad de Informática, Universidad Complutense de Madrid, Profesor Garcia Santesmases s/n, Madrid, Spain; 3 Centro de Biología Molecular ‘Severo Ochoa’, Universidad Autónoma de Madrid, Madrid, Spain; Kyushu Institute of Technology, Japan

## Abstract

A unique synaptic activity-responsive element (SARE) sequence, composed of the consensus binding sites for SRF, MEF2 and CREB, is necessary for control of transcriptional upregulation of the *Arc* gene in response to synaptic activity. We hypothesize that this sequence is a broad mechanism that regulates gene expression in response to synaptic activation and during plasticity; and that analysis of SARE-containing genes could identify molecular mechanisms involved in brain disorders. To search for conserved SARE sequences in the mammalian genome, we used the SynoR *in silico* tool, and found the SARE cluster predominantly in the regulatory regions of genes expressed specifically in the nervous system; most were related to neural development and homeostatic maintenance. Two of these SARE sequences were tested in luciferase assays and proved to promote transcription in response to neuronal activation. Supporting the predictive capacity of our candidate list, up-regulation of several SARE containing genes in response to neuronal activity was validated using external data and also experimentally using primary cortical neurons and quantitative real time RT-PCR. The list of SARE-containing genes includes several linked to mental retardation and cognitive disorders, and is significantly enriched in genes that encode mRNA targeted by FMRP (fragile X mental retardation protein). Our study thus supports the idea that SARE sequences are relevant transcriptional regulatory elements that participate in plasticity. In addition, it offers a comprehensive view of how activity-responsive transcription factors coordinate their actions and increase the selectivity of their targets. Our data suggest that analysis of SARE-containing genes will reveal yet-undescribed pathways of synaptic plasticity and additional candidate genes disrupted in mental disease.

## Introduction

Neuronal plasticity and memory formation require changes in gene expression that are triggered by synaptic activity. The nature and organization of this response is the subject of intense research, and a number of transcription factors (TF) have been identified in recent years as necessary for long-term memory consolidation and storage. The Ca^2+^/cAMP response element-binding protein (CREB) was initially identified as the main interlocutor in the dialogue between the synapse and the nucleus [1]. Later studies revealed the complexity of this process and implicated other transcription factors, including the serum response factor SRF [2], MEF2 [3] and Npas4 [4]. The availability of efficient methods for gene expression analysis has also contributed with a large collection of mRNAs, possible targets of these TF, whose expression is modulated by activity and experience [5], [6].

The large number of potential targets for these factors does not facilitate a model that clarifies how TF establish a coordinated response and regulate transcription for efficient remodeling of neuronal connections. The description of a 100 bp cis-regulatory enhancer element containing a cluster of CREB, MEF2 and SRF binding sites suggests a mechanism that might help to explain the selectivity and coordination of the activity-dependent transcriptional response. This sequence, termed SARE, was identified in the gene that encodes the activity-regulated cytoskeleton-associated protein (Arc) [7]. The SARE sequence is conserved in mammalian Arc regulatory regions; it is sufficient to drive a rapid transcriptional response following synaptic activation and to reproduce, both *in vitro* and *in vivo*, the endogenous Arc activation pattern [7]. Despite the novelty and potential repercussion of this finding, the study restricted the description of this sequence to the *Arc* gene and did not determine whether SARE appear in the regulatory regions of other genes, or the specificity of this sequence to the nervous system. We studied the broader implication of SARE sequences in the context of the response to neuronal activity, and validated SARE analysis as able to identify elements of synaptic plasticity. Using the *in silico* tool SynoR [8], we analyzed the SARE sequences conserved in the mammalian genome. Comparison of mouse and human genome sequences showed enrichment in conserved SARE clusters in the regulatory regions of genes that are expressed specifically in neural tissues, that are involved in neural development and homeostatic maintenance, and that encode mRNA targeted by FMRP. These data support the concept that SARE sequences are true transcriptional regulatory elements, responsible for the coordinated response of TF that convey information from the postsynaptic compartment to the nucleus. These findings might contribute to understanding the genetic causes of mental diseases linked to neuronal plasticity.

## Results and Discussion

**Figure 1:**
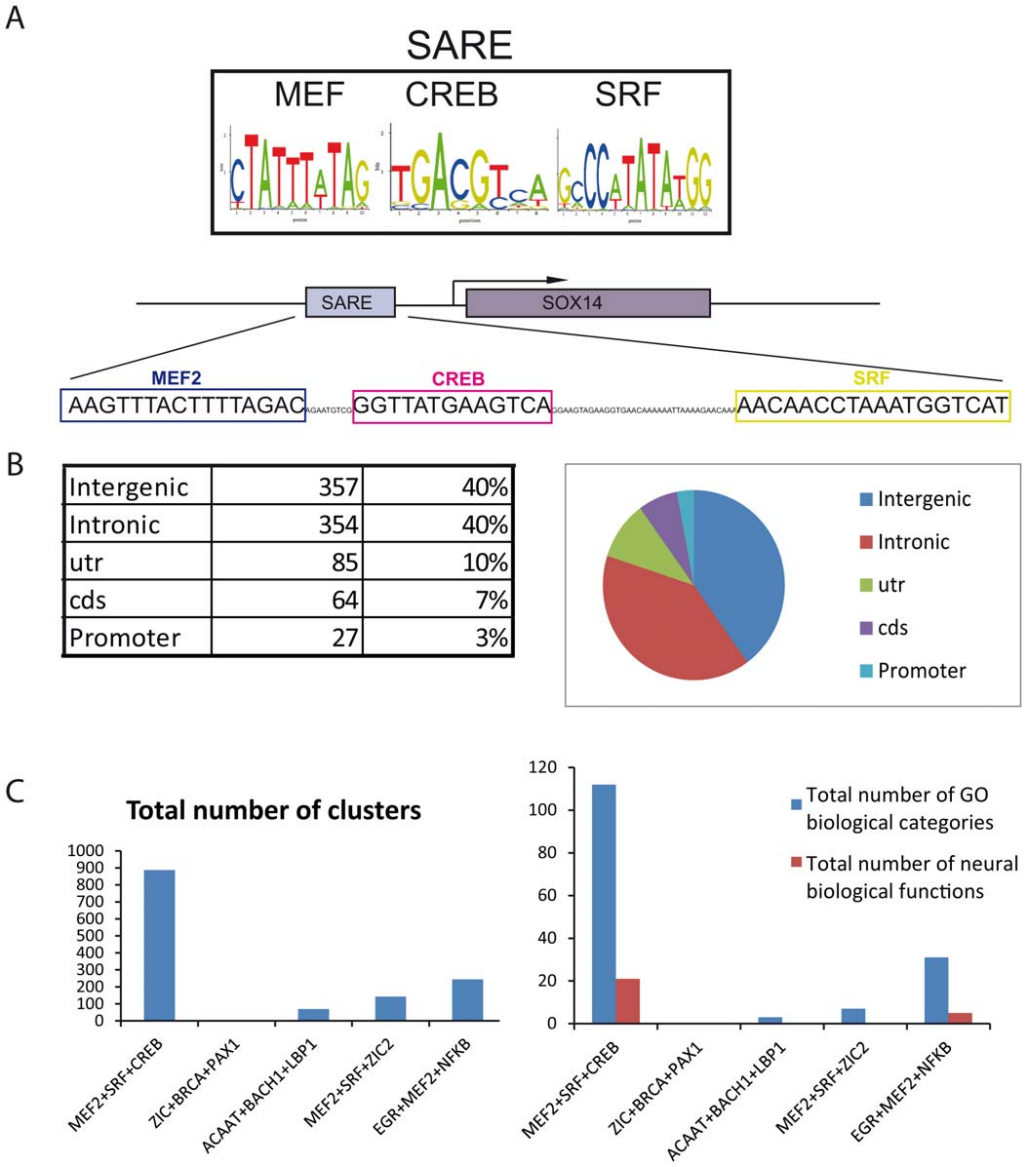
SARE sequences are found in intergenic and intronic genetic regions. Scheme showing consensus TFBS for SARE sequence and the SARE sequenced found in the promoter of *SOX14* (top). For the SynoR search, random relative position of the three TFBS was permitted. Table shows the number and percentages of SARE sequences classified according to their position within the genes and the diagram represents the distribution of each category.

We used SynoR to study the possible relationship between the SARE regulatory region and genes related to the nervous system [8], specifically those involved in synaptic activity and mental processes. We sought sequence regions containing clusters of the consensus binding sites for CREB, MEF2 and SRF (Fig. 1A) in the human genome, and compared them to the mouse genome to identify conserved sequences. Based on these criteria, we identified 887 genetic regions containing SARE sequences (Table S1 and data deposited in the SynoR tool, ID: s1219104005847). The SARE regions are assigned to the gene(s) of which they form part or to which they are proximal, and are classified as intergenic, intronic, utr (untranslated), cds (coding sequence), or promoter, depending on their position within the gene (Table S1 and Fig. 1B). Control searches for clusters containing combinations of other unrelated TFBS yield significantly less number of regions and were not enriched in neural biological functions (Fig. 1C and Experimental Procedures). The original SARE sequence of the Arc promoter is not identified in our search because it contains only half of the CREB binding site, and its MEF2 binding sequence shows 2 nucleotide mismatches compared to the consensus [7]. Binding site predictions for individual TF using matrix analysis can be conducted with the Match tool of TRANSFAC. We have validated the presence of the SARE cluster in more than ten of our candidates manually using this tool. These include *ATF3*, *CUX1*, *CUX2*, *FOXP1, FOXP2, HOMER1*, *LMPDH2*, *NRG1, NPAS4. NR4A1, PLXNA4 and SEMA6A.* Similarly, there might be additional SARE sequences not identified by this search because of the analysis procedure: it first identifies TFBS clusters in the human genome and subsequently searches for homology to this specific sequence in mice.

The analysis showed that SARE clusters were most abundant in intergenic and intronic regions (Fig. 1), potential areas for gene expression control. The list of SARE-containing genes showed genes with central roles in the nervous system such as NMDA (*Grin2a*, essential for excitatory synapses), *Robo2* (with major functions in axon guidance) and *Cutl/Cux2* (determinant for cerebral cortex layer II-IV) (examples in Table 1). Classification of genes containing SARE sequences at the GO categories using Toppfun application *(* http://toppgene.cchmc.org/ *“ToppFun”)* indicated that the processes potentially affected by SARE regulation are clearly related to the nervous system (Table S2). This analysis yielded several enriched GO categories, out of 112 significantly enriched GO biological processes, 21 (18,75%) of them related to neural functions (Table 2). All of these categories are specifically related to nervous system development and maintenance, and many showed significant greater enrichment than other categories (Table 2 and Table S2). In accordance with our hypothesis, this prevalence of neural functions supports potentially important, selective action of SARE-mediated mechanisms in the nervous system. Next, SARE containing genes were grouped into two main categories representing potential distal and proximal regulatory sequences: intergenic; and intragenic, cds, promoter and utr regions (Table S2) and GO analysis was performed separately with these two groups. Both groups showed similar enrichment in neural functions; therefore it did not favor proximal or distal regulatory regions as more relevant to plasticity.

**Table 1:** SARE regulation affects several aspects of neuron function.

Gene symbol	Name	Human gene ID	Type
**Synaptogenesis-Plasticity**			
SYNCRIP	synaptotagmin binding, cytoplasmic RNA interacting protein	10492	utr
HDAC5	histone deacetylase 5	10014	utr
HOMER1	homer homolog 1 (Drosophila)	9456	promoter
KALRN	kalirin, RhoGEF kinase	8997	utr
NCAM1	neural cell adhesion molecule 1	4684	intron
NRG1	neuregulin 1	3084	intron
NRXN1	neurexin 1	9378	intron
SCN3A	sodium channel, voltage-gated, type III, alpha subunit	6328	utr
SEPT7	septin 7	989	utr
**Citoesqueletal-synaptic proteins**			
ANK3	ankyrin 3, node of Ranvier (ankyrin G)	288	utr
CAMK1D	calcium/calmodulin-dependent protein kinase ID	57118	intron
MAP2K5	mitogen-activated protein kinase kinase 5	5607	intron
NR4A1	nuclear receptor subfamily 4, group A, member 1	3164	intron
RABGAP1L	RAB GTPase activating protein 1-like	9910	intron
RAPGEF2	Rap guanine nucleotide exchange factor (GEF) 2	9693	intron
RAPGEF6	Rap guanine nucleotide exchange factor (GEF) 6	51735	intron
RASSF8	Ras association (RalGDS/AF-6) domain family (N-terminal) member 8	11228	utr
RASGEF1C	RasGEF domain family, member 1C	255426	intron
RHOQ	ras homolog gene family, member Q	23433	utr
**Axon guidance and maintenance**			
CRIM1	cysteine rich transmembrane BMP regulator 1 (chordin-like)	51232	intron
DCC	deleted in colorectal carcinoma	1630	intron
EPHB1	EPH receptor B1	2047	promoter
NRG1	neuregulin 1	3084	intron
PLCXD3	phosphatidylinositol-specific phospholipase C, X domain containing 3	345557	intron
PLXNC1	plexin C1	10154	intron
PLXNA4	plexin A4	91584	intron
RGMA	RGM domain family, member A	56963	intergenic
ROBO1	roundabout, axon guidance receptor, homolog 1 (Drosophila)	6091	intergenic
SEMA6A	sema domain, TM and cytoplasmic domain, (semaphorin) 6A	57556	promoter
**Transcription factors**			
CUX1	cut-like homeobox 1	1523	intron
CUX2	cut-like homeobox 2	23316	intron
FOXP1	forkhead box P1	27086	intron
FOXP2	forkhead box P2	93986	intron
PAX6	paired box 6	5080	intron
PHOX2B	paired-like homeobox 2b	8929	utr
RUNX2	runt-related transcription factor 2	860	utr
SOX6	SRY (sex determining region Y)-box 6	55553	intron
ZIC2	Zic family member 2	7546	utr
ZNF292	zinc finger protein 292	23036	utr

Examples of genes containing SARE sequences according to function, extracted from the list of 827 genes identified.

**Table 2:** Classification of SARE-containing genes at GO indicated significant enrichment of processes related to the nervous system.

GO: Biological Process			
Rank	ID	Name	P-value
2	GO:0030182	neuron differentiation	1,76E-11
4	GO:0048699	generation of neurons	1,52E-10
5	GO:0022008	neurogenesis	2,28E-10
9	GO:0048812	neuron projection morphogenesis	2,87E-09
10	GO:0007409	axonogenesis	3,40E-09
13	GO:0048666	neuron development	6,43E-09
16	GO:0031175	neuron projection development	2,28E-08
28	GO:0007411	axon guidance	2,75E-07
31	GO:0007417	central nervous system development	3,49E-07
45	GO:0035295	tube development	9,04E-06
58	GO:0045664	regulation of neuron differentiation	1,27E-04
73	GO:0030900	forebrain development	1,74E-03
74	GO:0007420	brain development	1,75E-03
83	GO:0021772	olfactory bulb development	5,05E-03
84	GO:0031290	retinal ganglion cell axon guidance	5,46E-03
87	GO:0021889	olfactory bulb interneuron differentiation	6,39E-03
91	GO:0021988	olfactory lobe development	7,48E-03
96	GO:0007423	sensory organ development	1,29E-02
97	GO:0021537	telencephalon development	1,30E-02
111	GO:0001654	eye development	4,50E-02
112	GO:0021891	olfactory bulb interneuron development	4,54E-02

The analysis yielded 112 significantly enriched GO biological processes. The table shows the 21 categories with significantly high fold enrichment that are related to nervous system development and maintenance. Rank indicates the position within the list of the total 112 when ordered from higher to lower enrichment. Neural functions are ranked in the higher positions.

The analysis of genes containing the SARE cluster appeared an appropriate approach for identification of mechanisms of homeostasis, plasticity and activity-dependent remodeling in the nervous system. This study disclosed a large number of genes known to participate in plasticity and synaptogenesis (examples in Table 1); *Homer1* is an example in this category. *Homer* genes encode scaffolding proteins that bind Ca^2+^ signaling proteins and target them to their correct subcellular localization [9], [10]; they are essential for dynamic regulation of the synapse, synaptic plasticity, and spatial learning [11], [12]. Coincident expression of experience-triggered Homer and Arc proteins is found in hippocampal and cortical neurons [13], which supports simultaneous activation, as predicted by our analysis. We also identified axonal guidance molecules (Table 1 and Table S1), including *PlxnA4* and its ligand *Sema6A* [14] as molecules potentially regulated by SARE. The semaphorin and plexin receptor families, together with neuropilins, are crucial during nervous system development and homeostasis, and mark the pathway for axon growth [14]. These proteins also control synaptogenesis, axon pruning, the density and maturation of dendritic spines and are implicated in a number of developmental, psychiatric and neurodegenerative disorders [15]. As for the axon guidance cues, we found a number of genes that encode cytoskeletal remodeling molecules at the synapse (Table 1). For example, ankyrins link integral membrane proteins to the underlying spectrin-actin cytoskeleton; they have key roles in activities such as cell motility, activation, proliferation, contact, and maintenance of specialized membrane domains. They might be involved in bipolar disorder and other mental alterations [16].

Less anticipated were SARE-containing genes not previously implicated in plasticity or structural maintenance of the synapse; in this category, we found neuronal subtype-specific TF such as *Cux1* and *Cux2* [17], *Zic2* [18] and *Sox6* [19], [20] (Table 1 and Table S1). Cux TF expression is restricted to neurons of layers II-III and IV of the cerebral cortex. During development, Cux regulate dendritic branching, spine morphogenesis and synapse maturation [17]. Cux expression is maintained through adulthood, but nothing is yet known of their function in mature neurons. Whereas Cux functions could be associated with plasticity at the postsynaptic site [17], Zic2 might act on the presynaptic terminal, as it is associated with axon development in retinal ganglion cells; Sox6, in turn, is described as essential for neuronal differentiation [19], [20]. These observations suggest that activity-dependent mechanisms act on pathways specific to neuronal subtypes.

**Figure 2:**
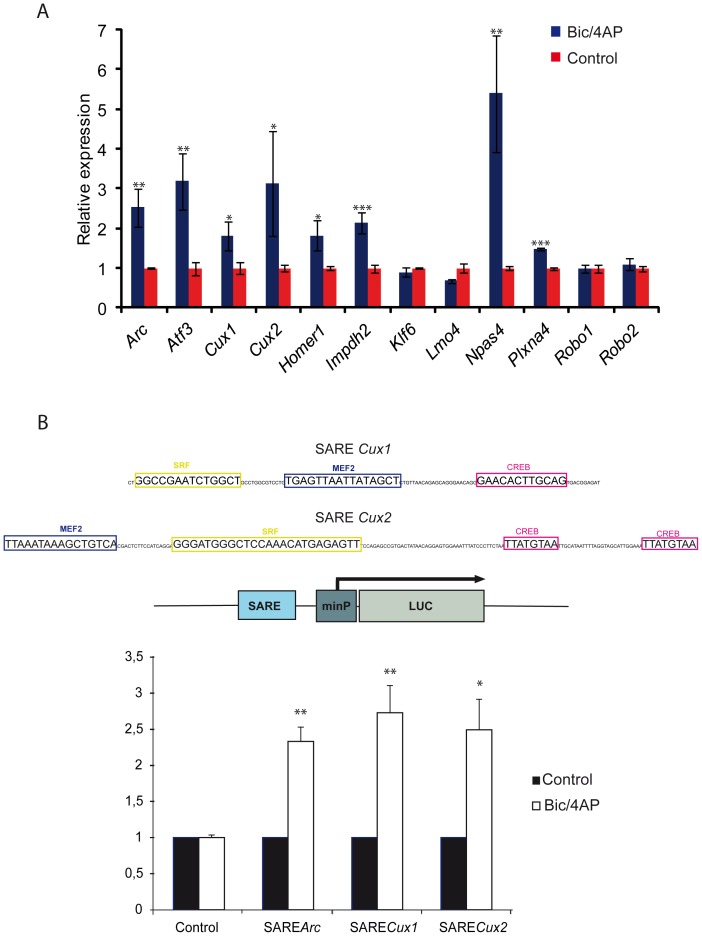
Up-regulation of the mRNA of SARE containing genes and promoter activation in response to neuronal activity. A) *Upregulation of SARE identified candidates in primary cortical neurons.* Primary neurons from E18 cortex were cultivated for 16 days before triggering neuronal activation with 4AP/bicuculline. RNA was obtained and transcript expression of SARE containing genes analyzed by quantitative real time RT-PCR (Q-PCR). Expression of each gene is normalized to the expression in control cells. Arc up-regulation demonstrated efficient neuronal activation. * indicates p<0,5 **<0,1, ***<0,05 (t-Student test). **B).** *Novel SARE sequences activate transcription in response to neuronal activity.* Mouse genomic sequence containing SARE regulatory regions (see below) corresponding to those identified for the *Cux1* and *Cux2* genes were cloned into the pGL4.23 luciferase vector (Promega). Neurons obtained from E18,5 cortex were co-transfected with the indicated firefly luciferase reporter construct and internal control Renilla luciferase plasmid at a ratio of 4∶1. Neuronal activity was trigered with 4AP/bicuculline before measuring transcription of the reporters. Relative expression of each reporter constructs was determined by normalizing the activity of each reporter to its activity on TTX treated neurons. Data represent mean and standar deviation of results obtained in three different experiments. * indicates p<0,01, **<0,001 (t-Student test).

To test the relevance of our findings and the predictive capability of our gene set to identify genes up-regulated upon neuronal activation we searched for experimental confirmation. Several studies of gene expression changes induced by neuronal activation have been reported and made useful available contributions. Many of them analyze the effects of the gabaergic inhibitor bicuculline to trigger neuronal excitatory response. We therefore compare the list of SARE containing genes with those of genes which expression was modify in studies analyzing the *in vivo* effects of infusion of bicuculline into the accessory olfactory bulb [21]; *in vitr*o bicuculline treatment of cortical cells [22]; and hippocampal neuronal cultures [23]. This allowed us to extend our validation to several neuronal types. In all three cases, the comparison revealed a highly significant enrichment between the SARE containing genes and those up-regulated upon neuronal activation, but none or of lower statistical significance, when compared to the list of genes that are down-regulated (Table 3). Several of the SARE genes, such as *Homer*, *Atf3*, *Klf6* and *Bdnf* are common to two or the three studies, and may represent a general pan-neuronal response, while unique ones might represent tissue-specific responses. These significant overlapping validate our results with external independent data. We next took the reverse approach and tested the predictive capability of our study by testing the expression of genes picked from our list upon neuronal activation. Cells from E18 mouse cortex were dissociated, neurons were cultivated and neuronal activity triggered using bicuculline [7]. RNA was obtained and transcript expression of twelve SARE containing genes, including Arc, analyzed by using quantitative real time RT-PCR (Q-PCR) (Fig. 2A). Up-regulation of Arc gene demonstrated efficient neuronal activation and, also expected, the levels of the S-isoform of *Homer1* were increased [24], [25]. Six more genes showed up-regulation when neurons were activated. *Atf3*, *Impdh2*, and *Npas4* up-regulation in cortical cells was in agreement with our own analysis of the raw data obtained from gene expression arrays reported by other investigators [22], and further confirmed our comparison with external sources (Table 3). Interestingly, up-regulation of *Cux1*, *Cux2*, and *PlxnA4,* genes not suspected to be regulated by activity, again confirmed the predictive capacity of our study. Four genes, *Lmo4*, *Robo1*, *Robo2* and *Klf6* did not show significant changes. This can be ascribed to the almost certain possibility of a number of false positive in our list, to the fact that other splicing variants might be affected, or to the possibility that subsets of genes may respond differently depending on the stimulus that triggers neuronal response.

**Table 3:** The number of SARE containing genes is significantly enriched on genes up-regulated upon bicuculline triggering of neuronal activity.

Comparative analysis with reported studies			
	SARE-Bic AOP ref [21]	SARE-Bic Hip ref [23]	SARE-Bic Ctx ref [22]
# of upregulated genes	(211 genes)	(90 genes)	(229 genes)
	*Atf3*	*Atf3*	*Arc*
	*Bdnf*	*Bdnf*	*Atf3*
	*Celf2*	*Edil3*	*Bdnf*
	*Clic4*	*Erc2*	*C10orf140*
	*Eif1*	*Inhba*	*Fbxo33*
	*Epb41l1*	*Ism1*	*Homer1*
	*Hdac5*	*Nkain2*	*Impdh2*
	*Hdac9*	*Odz1*	*Kcnj2*
	*Homer1*	*Pax1*	*Klf6*
Up-regulated genes	*Inhba*	*Ppfia2*	*Mex3b*
	*Klf6*		*Nefl*
	*Lingo1*		*Nhlh2*
	*Lmo4*		*Npas4*
	*Nemf*		*Nr4a1*
	*Pcdh17*		*Rasgef1b*
	*Ppm1b*		
	*Rcan2*		
	*Smg7*		
	*Tsc22d2*		
p-value	4,87E-05	0.00066422	0.003991
# of downregulated genes	(36 genes)	(44 genes)	(23 genes)
	*Bahcc1*	*Dock5*	
Down-modulated genes	*Ranbp2*	*Fam5b*	
		*Slit3*	
		*Trpc7*	
p-value	0.20867	0.042004	0.46753

Comparative analysis of the list of SARE containing genes with three independent studies reporting mRNA changes in gene expression in the accessory olfactory bulb [21]; cortical cells [22]; and hippocampal neuronal cultures [23]. The table shows the lists of the SARE genes that were found to be up-regulated (upper part), or down-modulated (lower part) in each study. P-values for random coincidence are shown. Not significance coincidence was observed when compared to genes that are down-regulated.

Next, the sequence corresponding to two of these SARE sequences were cloned upstream of a minimal promoter into vectors containing luciferase reporters to test their ability to activate transcription in response to neuronal activity. Cells from E18 mouse cortex were transfected with reporter constructs, neuronal activity triggered using bicuculline [7], and luciferase activity compared to control tetrodotoxin (TTX) treated neurons. These experiments demonstrated that these novel identified SARE sequences replicate the promoter activity of the SARE sequence corresponding to the Arc gene and significantly increase transcription upon depolarization (Fig. 2B).

Our analyses thus point to overlooked pathways that might participate in activity-dependent regulatory mechanisms and, by extension, suggests the identification of genes potentially linked to mental diseases caused by plasticity defects [26], [27], [28]. This is the case of genes reported as candidates for autism in which we found SARE sequences, such as, *NRXN1* and 2 [29], *FOXP1* [30], *FOXP2* [30], [31], *GRID2* [32], *KCNMA1* [33] and others (http://gene.sfari.org) [34] (see Table S1).

Validation of our prediction nonetheless required evaluation of true enrichment of genes involved in cognitive dysfunction. Fragile X syndrome (FXS) is a well-characterized form of autism, caused by loss of function of the Fragile X mental retardation protein (FMRP), which regulates local translation and plasticity at pre- and postsynaptic sites [28], [35]. Based on a recent extensive list of genes targeted by FMRP, from which the authors extract a stringent set of 842 reliable targets [36], we hypothesized that the list of SARE-regulated genes will be enriched in FMRP targets. Comparison of SARE-containing genes (including those containing SARE clusters at intergenic locations) with the stringent list of FMRP targets resulted in 70 genes common to both (8.5% overlap), an enrichment of biological relevance (p = 4.3909–13; see Methods) (Table 4). The relationship between the SARE-containing genes and FMRP targets thus strongly supports SARE involvement in activity-dependent regulation. In addition, it suggests that mutations in SARE or SARE-containing genes and pathways can contribute to mental retardation, autism spectrum disorders and other psychiatric diseases.

**Table 4:** FMRP targets containing SARE sequence genes.

FMRP targets containing SARE sequence				
*Gene Symbol*	*mm9 Symbol*	Entrez Gene ID	RefSeq ID	Description
*ANK3*	*Ank3*	11735	NM_146005.3	ankyrin 3, node of Ranvier (ankyrin G)
*APP*	*App*	11820	NM_007471.2	amyloid beta (A4) precursor protein
*ARID1B*	*Arid1b*	239985	NM_001085355.1	AT rich interactive domain 1B (SWI1-like)
*ATN1*	*Atn1*	13498	NM_007881.4	atrophin 1
*ATP2A2*	*Atp2a2*	11938	NM_001110140.2	ATPase, Ca++ transporting, cardiac muscle, slow twitch 2
*ATXN1*	*Atxn1*	20238	NM_009124.5	ataxin 1
*BAI1*	*Bai1*	107831	NM_174991.3	brain-specific angiogenesis inhibitor 1
*BCAN*	*Bcan*	12032	NM_007529.2	brevican
*BIRC6*	*Birc6*	12211	NM_007566.2	baculoviral IAP repeat-containing 6
*CADPS*	*Cadps*	27062	NM_012061.3	Ca++-dependent secretion activator
*CTNND2*	*Ctnnd2*	18163	NM_008729.2	catenin (cadherin-associated protein), delta 2
*CUX1*	*Cux1*	13047	NM_009986.3	cut-like homeobox 1
*CUX2*	*Cux2*	13048	NM_007804.2	cut-like homeobox 2
*DIP2B*	*Dip2b*	239667	NM_172819.2	DIP2 disco-interacting protein 2 homolog B (Drosophila)
*EPB41L1*	*Epb4.1l1*	13821	NM_001003815.2	erythrocyte membrane protein band 4.1-like 1
*FAM5B*	*6430517E21Rik*	240843	NM_207583.1	family with sequence similarity 5, member B
*FOXK2*	*Foxk2*	68837	NM_001080932.1	forkhead box K2
*GABBR1*	*Gabbr1*	54393	NM_019439.3	gamma-aminobutyric acid (GABA) B receptor, 1
*GNB1*	*Gnb1*	14688	NM_008142.3	guanine nucleotide binding protein (G protein), beta polypeptide 1
*GRIK3*	*Grik3*	14807	NM_001081097.2	glutamate receptor, ionotropic, kainate 3
*HDAC5*	*Hdac5*	15184	NM_001077696.1	histone deacetylase 5
*HIPK2*	*Hipk2*	15258	NM_001136065.1	homeodomain interacting protein kinase 2
*HIPK3*	*Hipk3*	15259	NM_010434.1	homeodomain interacting protein kinase 3
*IDS*	*Ids*	15931	NM_010498.2	iduronate 2-sulfatase
*KALRN*	*Kalrn*	545156	NM_001164268.1	kalirin, RhoGEF kinase
*KCND2*	*Kcnd2*	16508	NM_019697.3	potassium voltage-gated channel, Shal-related subfamily, member 2
*KCNH7*	*Kcnh7*	170738	NM_133207.2	potassium voltage-gated channel, subfamily H (eag-related), member 7
*KCNMA1*	*Kcnma1*	16531	NM_010610.2	potassium large conductance calcium-activated channel
*LINGO1*	*Lingo1*	235402	NM_181074.4	leucine rich repeat and Ig domain containing 1
*LRRC7*	*Lrrc7*	242274	NM_001081358.1	leucine rich repeat containing 7
*MAGI2*	*Magi2*	50791	NM_015823.2	membrane associated guanylate kinase, WW and PDZ domain containing 2
*MFHAS1*	*Mfhas1*	52065	NM_001081279.1	malignant fibrous histiocytoma amplified sequence 1
*MIB1*	*Mib1*	225164	NM_144860.2	mindbomb homolog 1 (Drosophila)
*MYT1L*	*Myt1l*	17933	NM_001093775.1	myelin transcription factor 1-like
*NCAM1*	*Ncam1*	17967	NM_001081445.1	neural cell adhesion molecule 1
*NFIX*	*Nfix*	18032	NM_001081981.1	nuclear factor I/X (CCAAT-binding transcription factor)
*NPAS2*	*Npas2*	18143	NM_008719.2	neuronal PAS domain protein 2
*NRXN1*	*Nrxn1*	18189	NM_020252.2	neurexin 1
*NRXN2*	*Nrxn2*	18190	NM_020253.2	neurexin 2
*NRXN3*	*Nrxn3*	18191	NM_172544.3	neurexin 3
*NTRK3*	*Ntrk3*	18213	NM_182809.2	neurotrophic tyrosine kinase, receptor, type 3
*ODZ2*	*Odz2*	23964	NM_011856.3	odz, odd Oz/ten-m homolog 2 (Drosophila)
*OGDH*	*Ogdh*	18293	NM_010956.3	oxoglutarate (alpha-ketoglutarate) dehydrogenase (lipoamide)
*PCDH7*	*Pcdh7*	54216	NM_001122758.1	protocadherin 7
*PCDH9*	*Pcdh9*	211712	NM_001081377.1	protocadherin 9
*PHACTR1*	*Phactr1*	218194	NM_198419.3	phosphatase and actin regulator 1
*PLXNA4*	*Plxna4*	243743	NM_175750.3	plexin A4
*PPARGC1A*	*Ppargc1a*	19017	NM_008904.1	peroxisome proliferator-activated receptor gamma, coactivator 1 alpha
*PRICKLE2*	*Prickle2*	243548	NM_001081146.1	prickle homolog 2 (Drosophila)
*PTCH1*	*Ptch1*	19206	NM_008957.2	patched homolog 1 (Drosophila)
*PTPRG*	*Ptprg*	19270	NM_008981.3	protein tyrosine phosphatase, receptor type, G
*PUM2*	*Pum2*	80913	NM_030723.1	pumilio homolog 2 (Drosophila)
*R3HDM2*	*R3hdm2*	71750	NM_027900.3	R3H domain containing 2
*RAPGEF2*	*Rapgef2*	76089	NM_001099624.2	Rap guanine nucleotide exchange factor (GEF) 2
*SASH1*	*Sash1*	70097	NM_175155.4	SAM and SH3 domain containing 1
*SLITRK5*	*Slitrk5*	75409	NM_198865.1	SLIT and NTRK-like family, member 5
*SMARCA2*	*Smarca2*	67155	NM_011416.2	SWI/SNF related, matrix associated, actin dependent regulator of chromatin
*SMG1*	*2610207I05Rik*	233789	NM_001031814.1	SMG1 homolog, phosphatidylinositol 3-kinase-related kinase (C. elegans)
*SPRED1*	*Spred1*	114715	NM_033524.2	sprouty-related, EVH1 domain containing 1
*TANC2*	*Tanc2*	77097	NM_181071.3	tetratricopeptide repeat, ankyrin repeat and coiled-coil containing 2
*TCF4*	*Tcf4*	21413	NM_013685.2	transcription factor 4
*TRIO*	*Trio*	223435	NM_001081302.1	triple functional domain (PTPRF interacting)
*TRIP12*	*Trip12*	14897	NM_133975.4	thyroid hormone receptor interactor 12
*VPS13D*	*Vps13d*	230895	NM_001128198.1	vacuolar protein sorting 13 homolog D (S. cerevisiae)
*ZEB2*	*Zeb2*	24136	NM_015753.3	zinc finger E-box binding homeobox 2
*ZNF365*	*Zfp365*	216049	NM_178679.2	zinc finger protein 365
*ZNF462*	*Zfp462*	242466	NM_172867.3	zinc finger protein 462
*ZNF521*	*Zfp521*	225207	NM_145492.3	zinc finger protein 521
*ZNF536*	*Zfp536*	243937	NM_172385.2	zinc finger protein 536
*ZNF827*	*Zfp827*	622675	NM_178267.3	zinc finger protein 827

The list of genes identified as containing SARE sequences was compared to a list of 842 reliable FMRP targets. This resulted in an overlap of 70 genes common to both lists. This represent a significant enrichment of p = 4.39096.93e-13, far from the expected random distribution of coincidences between the genome and the mouse nervous system transcriptome (see Experimental Procedures).

Correct function of nervous system networks and subnetworks is possible thanks to the extraordinary spatial and temporal coordination of gene expression that is guided by the TF subset expressed by each neuronal population. Our findings suggest that cooperation between CREB, SRF, and MEF2 transcription factors at the SARE region is one of the precisely regulated mechanisms that govern the transcriptional program of activated neurons. This transcriptional cooperation might also apply to other TF to initiate an appropriate, specific transcriptional response in other biological processes. This study also highlights the value of the development and use of computational tools and databases for the comprehensive analysis of biological events. We identified a subset of genes whose transcription is potentially regulated by the SARE cluster after synaptic activation. Most of these genes are directly related to nervous system development and maintenance; several of them are reported at the synapse, some are mutated in human mental disorders, and many form part of FMRP-regulated mechanisms. The identification and functional analysis of SARE-containing genes provided here is thus a useful for implicating new candidate genes in plasticity, memory, and mental retardation, and suggests new approaches to the study of mental disorders in which synaptic activity might have a central role.

## Methods

### Genome Sequence Analysis

*In silico* analyses were performed using SynoR (Identifying synonymous regulatory elements in vertebrate genomes), a tool described by Ovcharenko and Nobrega [8]. SynoR is available at the National Center for Biotechnology Information (NCBI) DCODE.org Comparative Genomics Developments (http://synor.dcode.org/), and performs *de novo* identification of synonymous regulatory elements (SRE) using known patterns of transcription factor binding sites (TFBS) in active regulatory elements (RE) as seeds for genome scans. The search was performed on the human genome assembly (hg18; July 2007 NCBI Build 36.1) and compared to the mouse genome assembly (mm9; July 2007NCBI Build 37). ECBR Browser performs whole genome Blastz-based alignments using the TFBS data of the transcription factors under study from the TRANSFAC Professional database. The TFBS studied were those of CREB (CREB_01, CREBP1_01, CREBP1CJUN_01, CREB_02, CREB_Q2, CREB_Q4, CREBP1_Q2, CREB_Q3, CREB_Q2_01, CREB_Q4_01, CREBATF_Q6), MEF2 (MEF2_01, MEF2_02, MEF2_03, MEF2_04, MEF2_Q6_01) and SRF (SRF_01, SRF_Q6, SRF_C, SRF_Q4, SRF_Q5_01, SRF_Q5_02). The maximum distance between two adjacent TFBS was set at 125 base pairs. Random relative position of TFBS was allowed. To test for the significance of the results, we performed analysis of several TFBS combinations. A search for clusters combining TFBS for ZIC2, BRCA and PAX1 yields only 1 cluster indentified by SynoR, combination of ACAAT, Bach1, Lbp1 gives 50 clusters. Combination of TF related to the nervous system, EGR1, MEF2, NF-kB results on 244 clusters identified. Substitution of any of the TFBS from our particular search of MEF2, CREB and SRF significantly decreased the number of identified clusters. For example, substitution of CREB for ZIC2, i.e. search for MEF2, SRF and ZIC2 gives 142, compared to 842.

### Gene Annotation and Analysis of Gene Ontolog

The list of SARE genes obtained from SynoR was updated using Toppfun application from Toppgene suit Cincinnati Children’s Hospital Medical Center *(* http://toppgene.cchmc.org/ *“ToppFun”)* and manually curated. A small number of genes were not annotated in NCBI but appeared annotated in other data bases (Shaded genes on STableI). GO analysis was performed using Toppfun shown in table S2. Similar significant enrichment of nervous system related functions of GO categories were obtained from the SynoR application.

### Gene Expression Data Sets of Bicuculline Treatments

Experimental datasets were obtained from [23]–[24] as processed datasets. Unprocessed data from [22] was analyzed using GeoR2 (http://www.ncbi.nlm.nih.gov/geo/geo2r/). Gene annotation was updated using Toppfun application for all three datasets.

### Primary Neurons Culture and Bicuculline/4-amino-pirydine Induction

Neurons from E18 embryo cortex were trypsinized using 0,25 µg/ml trypsin (SIGMA-Aldrich) in EBSS (Gibco, Invitrogen, Carlsbad, CA) 3.8% MgSO4 (Sigma-Aldrich, St. Louis, MO), penicillin/streptomycin (Gibco, Invitrogen, Carlsbad, CA). The reaction was stopped and cells were mechanical dissociated in EBSS media complemented with 0,26 mg/ml Trypsin inhibitor, 0,08 mg/ml DNAse, and 3,8% MgSO4 heptahydrate (all from Sigma-Aldrich, St. Louis, MO). Dissociated cells were seeded onto 24 well Poly-D-Lys (Sigma-Aldrich, St. Louis, MO) coated plates in neurobasal media supplemented with B27 complement 1×, glutamax 1× and penicillin/streptomycin (Gibco, Invitrogen, Carlsbad, CA). 500 µl media were replaced every 2 days until 16 days (DIV 16). 12 h before inducing neuronal activity cells were incubated with 2 mM tetrodotoxin (TTX) (Alomone-labs, Jerusalem, Israel). Then media was replaced with media containing with 4-aminopyridine (4AP) 100 µM, strychnine 1 µM, glycin 100 µM and bicuculline 30 µM (Sigma-Aldrich, St. Louis, MO ), and RNA was extracted after 5 h using the Qiagen RNeasy Kit as described in the manufacture handbook (Qiagen).

### Luciferase Reporter Assays

Mouse genomic sequence containing SARE regulatory regions (see below) corresponding to those identified for the Cux1 and Cux2 genes were cloned into the pGL4.23 luciferase vector (Promega). The SARE sequence for Cux1 is CTGGCCGAATCTGGCTGCCTGGCGTCCTGTGAGTTAATTATAGCTCTGTTAACAGAGCAGGGAACAGGGAACACTTGCAGTGACGGAGAT. The sequence for Cux2 TTAAATAAAGCTGTCACGACTCTTCCATCAGGAGGGATGGGCTCCAAACATGAGAGTTTCCAGAGCCGTGACTATAACAGGAGTGGAAATTTATCCCTTCTAATTATGTAATTGCATAATTTTAGGTAGCATTGGAAATTATGTAA. E18,5 neuronal cells cultured for 8 days were co-transfected with the corresponding firefly luciferase reporter constructs and internal control Renilla luciferase plasmid, at a ratio of 4∶1 using lipofectamine 2000 (Invitrogen). Neuronal response was trigger using bicuculline/4-amino-pirydine as described above. Control cells Luciferase and Renilla activity was measure using the Dual-Luciferase Reporter Assay System (Promega) and following the manufacture protocol. Relative expression of each reporter construct was determined by normalizing the ratio of reporter activity to the activity on TTX treated neurons.

### Q-PCR Analysis

1 µgr of total RNA was reverse transcribed with random primers (Invitrogen-Life Technologies, Carlsbad CA) and the superscript reverse transcriptase (New England BioLabs, Beverly, MA). PCR reaction mixtures containing DNA Master Sybr green I mix (Applied Biosystems, Foster City, CA) were incubated at 95°C for 5 min followed by 40 PCR cycles (5 s at 95°C, 45 s at 60°C, 90 s at 68°C) in an Abi-prism 7000 detector (Applied Byosystems). Primers for *Robo 1*(forward GACCTGATCGTCTCCAAAGGA; reverse TTGTCGGTCTCCACTCTTTCC); *Robo2* (forward TGATGGATCTCGTCTTCGTCA; reverse GTCGGCCCTCTGCTTTACAG); *Cux1* (forward GGGGCTTTTTATCTGCCATC; reverse CCCCCTTCCTGGTTTAAGAAG); *Cux2* (forward CTGTCCTTCATTGCACAACC; reverse TTCGGAGGTGGACTTGAAAC); *Atf3* (forward TCCTGGGTCACTGGTATTTG; reverse ATGGCGAATCTCAGCTCTTC); *Impdh2* (forward CTCCAAAGATGCCAAGAAGC; reverse TGGGAAGAGTCCAAAACCAC); *Npas4* (forward ACCTGAGCAAGGATTTGGTG; reverse TTGGTGTCAGCTGTTCTTGG); *Klf6* (forward CACCCACGACCAAATTTACC; reverse TGGAAATGACGGAGGAACTC); *Homer1-S* (forward GAAAGCTTTACCACAGGCCTAC; reverse TCAATGCTAACAGGCTCGTG); *Arc* (forward TGTTGACCGAAGTGTCCAAG; reverse AAAGACAGGCCTTGATGGAC), *Lmo4* (forward ACATTGGCACGTCCTGTTAC; reverse TCACTTGCAGGAATCGACTG) and *PlxnA4* (forward TGAGGACAACCCCAAGTGTTA; reverse ACGCGATCAGCCTGTTTTCT) were tested. The results were normalized as indicated by the parallel amplification of *β-actin* (forward GGCTGTATTCCCCTCCATCG; reverse CCAGTTGGTAACAATGCCATGT).

### Statistical Analysis

Probability of overlap between the FMRP target gene, and the SARE-containing gene lists was based on a binomial function, considering the size of the human genome as 28000 genes; the mouse nervous system transcriptome as 12000 transcripts and the total number of all genes associated to one or more SARE cluster is 827. We calculated the density function that describes the probability of having a number of genes within the transcriptome, with the binomial *X = B*(n_1_ = 827, p = 12/27) We then obtained the density function Y, which describes the probability of having a number of coincidences between both lists, with a new binomial 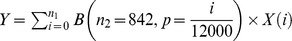 (*n* = total number of FMRP targets). This probability distribution yielded a p value of 4.3909e-13 for Y(70) (for 70 coincidences). Equal analysis was performed to calculate the significance of overlapping between datasets from experimental data of bicuculline modulated genes and the SARE containing genes list.

## Supporting Information

Table S1 **SARE sequences conserved in human and mouse assigned to genes according to proximity.** Using SynoR, we searched for regions containing clusters of the consensus binding sequences for SRF, MEF2 and CREB in the human genome and compared it to the mouse genome to identify conserved sequences. Based on these criteria, we identified 887 genetic regions with conserved SARE sequences that are assigned to the proximal genes: 530 clusters were found on intragenic regions and 357 in intergenic, (data deposited in the SynoR tool, ID n° s1219104005847). Additional tab (OtherTFclusters) show results from the analysis of other TFBS combinations and graphics showing the relative low number of clusters identified and their lower relation to neuronal functions.
(XLSX)

Table S2 **Gene Ontology analysis of SARE-containing genes.** GO analysis of all genes containing the SARE cluster performed by Toppfun application. Additional tab on table shows the 21 GO categories out of the total 112 enriched GO categories of biological functions related to nervous system and the same analysis performed on subsets of SARE containing genes grouped as intergenic (distant regulated genes) or intronic, promoter, CDS and utr (closely regulated genes). Most of the functions seem to be regulated by both distant and close SARE, but some of them are specific to each category (light shaded blue).
(XLSX)
